# Analysis of Disparity Information for Depth Extraction Using CMOS Image Sensor with Offset Pixel Aperture Technique †

**DOI:** 10.3390/s19030472

**Published:** 2019-01-24

**Authors:** Byoung-Soo Choi, Jimin Lee, Sang-Hwan Kim, Seunghyuk Chang, JongHo Park, Sang-Jin Lee, Jang-Kyoo Shin

**Affiliations:** 1 School of Electronics Engineering, Kyungpook National University, Daegu 41566, Korea; bschoi@ee.knu.ac.kr (B.-S.C.); jmLee@ee.knu.ac.kr (J.L.); shkim7@knu.ac.kr (S.-H.K.); 2 Center for Integrated Smart Sensors, KAIST, Daejeon 34141, Korea; schang71@kaist.ac.kr (S.C.); parkjh20@kaist.ac.kr (J.P.); sjlee82@kaist.ac.kr (S.-J.L.)

**Keywords:** CMOS image sensor, disparity information, depth extraction

## Abstract

A complementary metal oxide semiconductor (CMOS) image sensor (CIS), using offset pixel aperture (OPA) technique, was designed and fabricated using the 0.11-µm CIS process. In conventional cameras, an aperture is located on the camera lens. However, in a CIS camera using OPA technique, apertures are integrated as left-offset pixel apertures (LOPAs) and right-offset pixel apertures (ROPAs). A color pattern is built, comprising LOPA, blue, red, green, and ROPA pixels. The disparity information can be acquired from the LOPA and ROPA channels. Both disparity information and two-dimensional (2D) color information can be simultaneously acquired from the LOPA, blue, red, green, and ROPA channels. A geometric model of the OPA technique is constructed to estimate the disparity of the image, and the measurement results are compared with the estimated results. Depth extraction is thus achieved by a single CIS using the OPA technique, which can be easily adapted to commercial CIS cameras.

## 1. Introduction

Complementary metal oxide semiconductor (CMOS) image sensors (CISs) are widely used in various products, such as mobile phones, digital single-lens reflex cameras, and closed-circuit television systems [1,2,3]. CISs have several advantages, including high integration, low power consumption, and high frame rates. Additionally, they have been developed for imaging systems. In a conventional CIS camera, only two-dimensional (2D) images without depth information can be obtained. To obtain depth information, time-of-flight, stereo vision, and structured light techniques have been used [4,5,6,7,8,9,10,11]. These techniques, however, have disadvantages. A high-power external light source is required for the time-of-flight technique, and the stereo vision technique usually requires more than a single camera. Furthermore, the structured light technique requires a high-cost hardware system.

In this study, a CIS using offset pixel aperture (OPA) technique is developed, and its performance is evaluated. Three-dimensional (3D) information with 2D color and disparity information related to depth can be obtained using a single CIS without an external light source. This paper is an extended version of a previous paper [12]. A CIS using OPA technique is based on the depth-from-disparity method. In this method, the disparity of an image increases with the distance between the camera and the object. Several techniques based on the depth-from-disparity method have been developed to obtain images using disparity information [13,14,15,16]. The depth-from-disparity method was previously used for phase detection auto-focusing (PDAF) [17,18]. However, the depth resolution of the proposed OPA technique could be better than that of the PDAF technique because the offset of two apertures in the proposed OPA technique is larger. Furthermore, the proposed OPA technique uses no color filter, resulting in higher sensitivity compared to the PDAF technique. The concept of the OPA technique was previously proposed [19] and the hardware implementation for depth extraction was developed [20].

In this paper, disparity information for depth extraction using CIS with OPA technique is analyzed based on the theory of a mathematical model. In addition, the effects of aperture offset in the OPA of the pixels are investigated using optical simulation based on the finite-difference time-domain method. The OPA was designed and fabricated using the first metal layer in the CIS process. The aperture offset is optimized via optical simulation. Based on simulation results, a CIS using OPA technique was fabricated, and the performance of the fabricated CIS is evaluated. It uses small pixels, based on four transistors active pixel sensors with the pinned photodiode, and it can be easily adapted to high-resolution conventional CISs. The geometric model of the OPA technique is constructed to estimate the disparity of the image, and its operating principle is explained. In the measurement result, the disparity information is obtained from the left-OPA (LOPA) and right-OPA (ROPA) channels of the CIS. The measurement result with disparity, according to distance, are compared with results estimated from the geometric model. The study provides a useful reference for further research in depth extraction and is useful in the implementation of 3D camera systems for gesture recognition, motion detection, and object reconstruction.

## 2. Operating Principle

**Figure 1:**
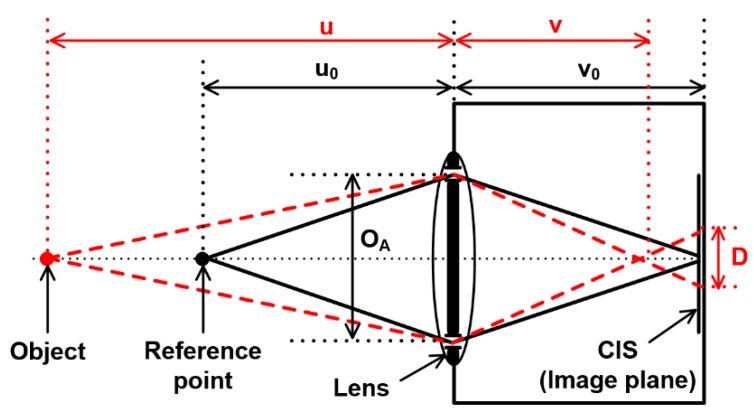
Geometric model for the depth-from-disparity method.

Depth-from-disparity is an attractive method of depth extraction. Stereo vision is a typical technique based on the depth-from-disparity method. The OPA technique in this paper is also based on the depth-from-disparity method. In this method, a pair of 2D images obtained from two viewpoints are used for depth extraction. The amount of disparity depends on the distance to the surface of the focus point and the characteristics of the camera lens. The disparity of the image increases with the distance between the camera lens and the object. The geometric model for the depth-from-disparity method is shown in Figure 1, and its principle is explained as follows:

(1) $$\frac{1}{u}+\frac{1}{v}=\frac{1}{f}$$

(2) $$v=\frac{fu}{u-f}$$

(3) $$v_{0}=\frac{{fu}_{0}}{u_{0}-f},$$

where *u* is the distance between the focused point of a object and a lens, *v* is the distance between the lens and the focused plane, and *f* is the focal length of the lens. The model has two holes, and the baseline (*O_A_*) is a distance between the holes. The disparity (*D*) is related to the parameters of the model and defined in the following equations:

(4) $$v-v_{0}=\frac{f^{2}\left(u-u_{0}\right)}{\left(u-f)(f-u_{0}\right)}$$

(5) $$D=\frac{(v-v_{0})O_{A}}{v}=\frac{f^{2}\left(u-u_{0}\right)}{u(f-u_{0})}\cdot \frac{O_{A}}{f}.$$

**Figure 2:**
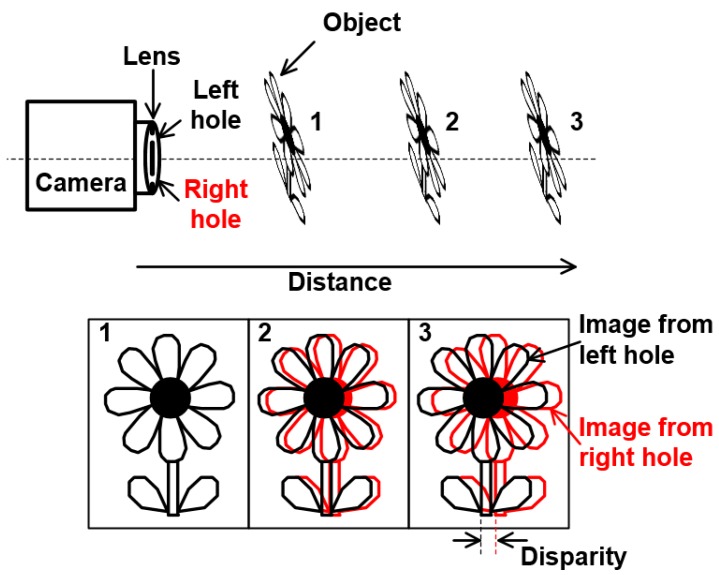
Disparity images from the left and right holes.

This equation indicates that the image has no disparity at *u* = *u*_0_, and the disparity is proportional to distance. The disparity is also proportional to distance between the two holes. However, the depth extraction is more difficult and the possibility of a false match increases as the distance between the two holes increases. Figure 2 shows the disparity of the captured images from the left and right holes. When these images are distinguished, the disparity can be calculated by the differences of between two correlated points of the images from the left and right holes.

**Figure 3:**
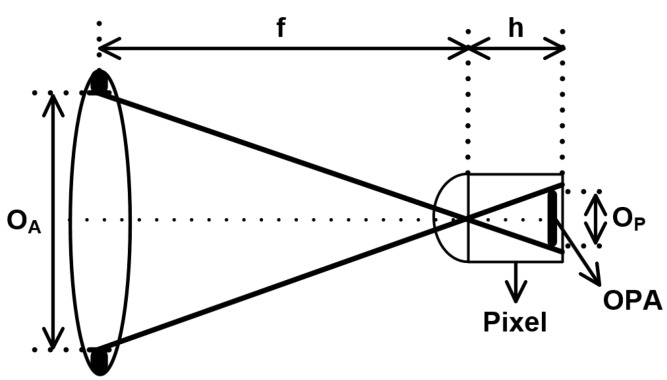
The geometric model of the OPA technique.

The geometric model of the OPA technique is shown in Figure 3. The OPA in the pixel acts as two holes in the lens. The equivalent F-number (*F_P_*) of the OPA technique is defined as the following equation:

(6) $$F_{P}=\frac{f}{O_{A}}=\frac{h}{O_{P}},$$

where *O_A_* and *f* are defined in Equation (5). *h* is the height of the pixel and *O_P_* is offset of the OPA. The disparity related to the parameters of the geometric model of the OPA technique is redefined from Equation (5):

(7) $$D=\frac{f^{2}\left(u-u_{0}\right)}{u\left(f-u_{0}\right)F_{P}}=\frac{f^{2}\left(u-u_{0}\right)}{u\left(f-u_{0}\right)}\cdot \frac{O_{P}}{h}.$$

The disparity is related to the offset of the OPA and the height of the pixel. To increase disparity, the offset of the OPA should be large, or the height of the pixel should be low. However, the height of the pixel is fixed by the CIS process and the offset is limited to pixel size. Therefore, optimization of the offset is necessary to increase the disparity.

**Figure 4:**
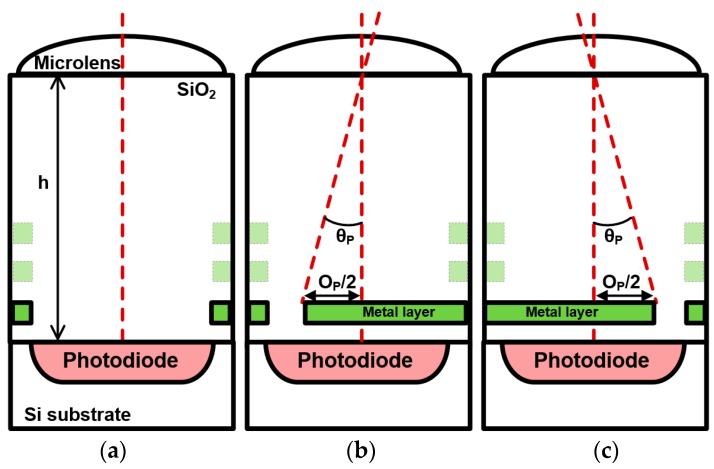
Vertical structures of (**a**) blue, red, green, (**b**) LOPA, and (**c**) ROPA pixels.

The vertical structure of the blue, red, and green pixels without the OPA is shown in Figure 4a, and the vertical structures of the LOPA and ROPA pixels are shown in Figure 4b,c, respectively. The vertical structure comprises a microlens, silicon oxide (SiO_2_), metal layers, a photodiode, and a silicon (Si) substrate. In the LOPA and ROPA pixels, most of the light incident on the photodiode has an incidence angle of *θ_P_* owing to the aperture offset (*O_P_*/2). The pixel apertures of the LOPA and ROPA were designed in the first metal layer using a CIS process where the light is focused on the first metal layer with a microlens. The second and third metal layers are used for signal lines in the pixels. The radius of curvature for the microlens and its height from the first metal layer are elaborately considered and modeled. The LOPA and ROPA are based on white pixel, which does not have a color filter. The white pixel is ideal for the OPA technique to ensure higher sensitivity.

**Figure 5:**
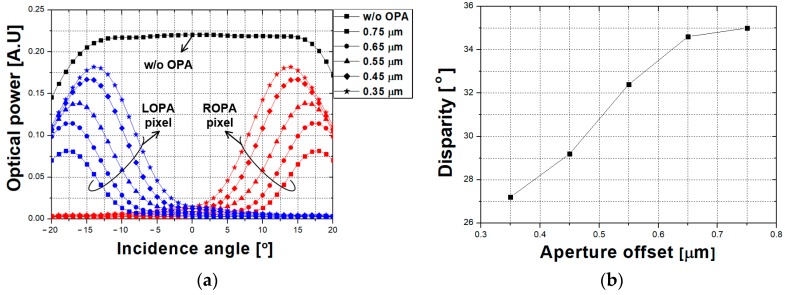
Optical simulation results that demonstrate (**a**) the variations in optical power with the incidence angle as a function of the aperture offset and (**b**) the disparity according to the aperture offset.

Figure 5a presents the optical simulation results that demonstrate variations in optical power with the incidence angle as a function of the aperture offset. For implementing optical simulation using the finite-difference time-domain method, consideration is paid to the refractive index, the heights of the microlens and active pixel sensor, and the position of the layers of the active pixel sensor. As the aperture offset increases, the peak point of the optical power is shifted. However, sensitivity-related optical power at the peak point decreases with the aperture offset, because the amount of transmitted light decreases. Disparity according to the aperture offset is shown in Figure 5b. The aperture offset is optimized at 0.65 µm, and the CIS using the OPA technique for depth extraction was fabricated, based on optical simulation.

## 3. Design and Fabrication

**Figure 6:**
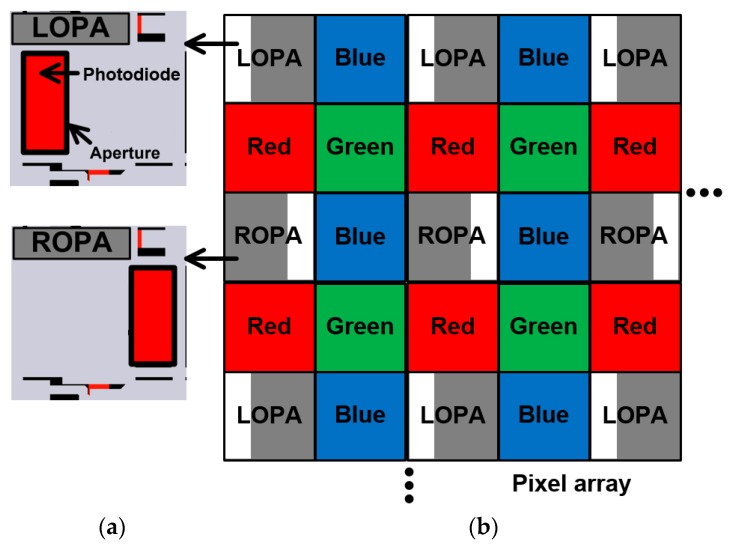
(**a**) Layouts of the LOPA and ROPA pixels and (**b**) color pattern of the pixel array.

Figure 6a shows the layouts of the LOPA and ROPA pixels. The active pixel sensor is based on four transistors with a pinned photodiode. The incident light is focused at the center of the pinned photodiode and is converted to an electrical signal. The active pixel sensor is suitable because of its small size, high sensitivity, and low dark current. The size of unit pixel is as small as 2.8 × 2.8 µm^2^, and the fill factor of the pixel is 39%. The resolution of pixel array is 1632 (horizontal, H) × 1124 (vertical, V) with the optical block. The pixel was designed using the 0.11-µm 2-poly 4-metal CIS process. In the LOPA and ROPA pixels, the incident light with a high incidence angle is transmitted by the LOPA and ROPA. The disparity information can be obtained from the aperture in the LOPA and ROPA pixels, because the LOPA and ROPA pixels produces different incidence angles of the transmitted light.

The color pattern of the pixel array is shown in Figure 6b. The color pattern comprises the LOPA, blue, red, green, and ROPA pixels. A unit pattern comprises 4 × 4 pixels. The blue, red, and green pixels with color filters are used to obtain color images, and the LOPA and ROPA pixels are used to obtain disparity images.

**Figure 7:**
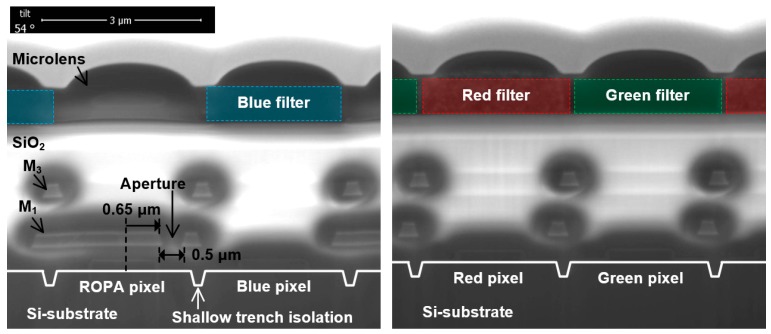
Structures of the fabricated pixels obtained by the scanning electron microscopy.

Figure 7 shows the structures of the fabricated pixels obtained by the scanning electron microscopy. The view is vertically tilted 54°. The structures comprise microlens, color filters, SiO_2_, third metal layers (M_3_), first metal layers (M_1_), Si substrates, and shallow trench isolations. There is no color filter in the ROPA pixel based on the white pixel.

**Figure 8:**
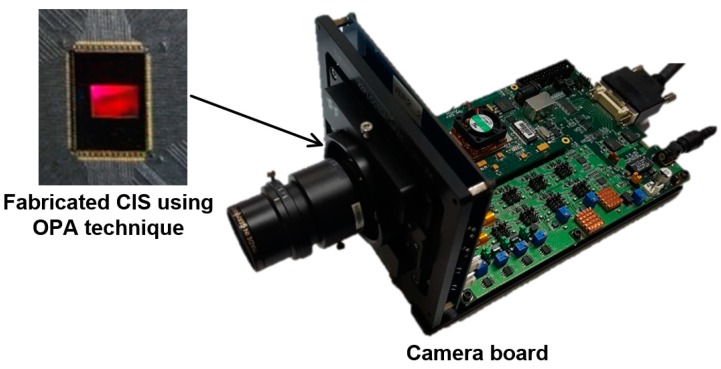
Camera board with the fabricated CIS using the OPA technique.

The camera board with the fabricated CIS using the OPA technique is shown in Figure 8. The performance of the fabricated CIS is evaluated using this camera board. The fabricated CIS comprises a pixel array with the color patterns, driving circuits (including the vertical and horizontal scanners), column-parallel readout circuits, bias circuits, and other components. A voltage of 1.5 V is used for the analog and digital part, and one of 3.3 V is used for the analog part. The frame rate and exposure time for the measurement are 17 frames per second (fps) and 59 ms, respectively. The fabricated CIS is summarized in Table 1.

## 4. Measurement Results and Discussion

**Figure 9:**
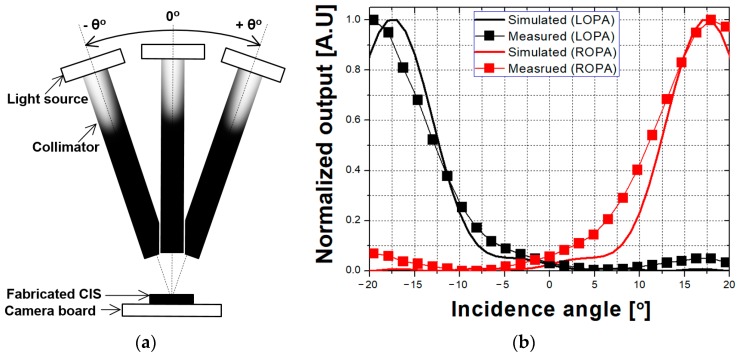
(**a**) Measurement system used to evaluate performance of the fabricated CIS using the OPA technique according to the incidence angle and (**b**) comparison of the simulation and measurement results.

Figure 9a shows the measurement system used to evaluate the performance of the fabricated CIS using the OPA technique according to the incidence angle. In the measurement system, a collimator was used to produce parallel light, and the incidence angle was changed from −20° to 20°. The range of incidence angle was determined by considering an F-number of 1.4 with a maximum incidence angle of 19.65° when a camera lens was used to obtain the images. The measurement was performed under dark conditions, and the output of the camera without the lens was measured.

The comparison of the simulation and measurement results is shown in Figure 9b. The output of the simulation results is based on Figure 5 when the aperture offset is 0.65 µm. The output of the measurement results was averaged over 100 frames to suppress the influences of temporal random noise. The output of the simulation and measurement results was normalized to a range from 0 to 1. For both simulation and measurement results, the incidence angles at the peak points of the outputs in the LOPA and ROPA pixels are shifted by more than 17° from 0°. The incidence angle at the peak point is determined by *θ_P_*-related aperture offset, as shown in Figure 4. These results confirm that light with a high incidence angle is transmitted in the LOPA and ROPA pixels. The measurement results agreed well with the simulation results, but with slightly different output. The disparities of the simulation and measurement results are 35.2° and 36.9°, respectively. The difference in the disparities between simulation and measurement results is 1.7°, caused by different characteristics between the ideal microlens model for the simulation and the fabricated microlens. Additionally, the incidence angle at the cross point of outputs in the LOPA and ROPA pixels is slightly shifted by −1.4° from 0° in the measurement result, because microlens is shifted, owing to the overlay in the fabrication of CIS process. To obtain better performance, an improvement in the microlens process is necessary.

**Figure 10:**
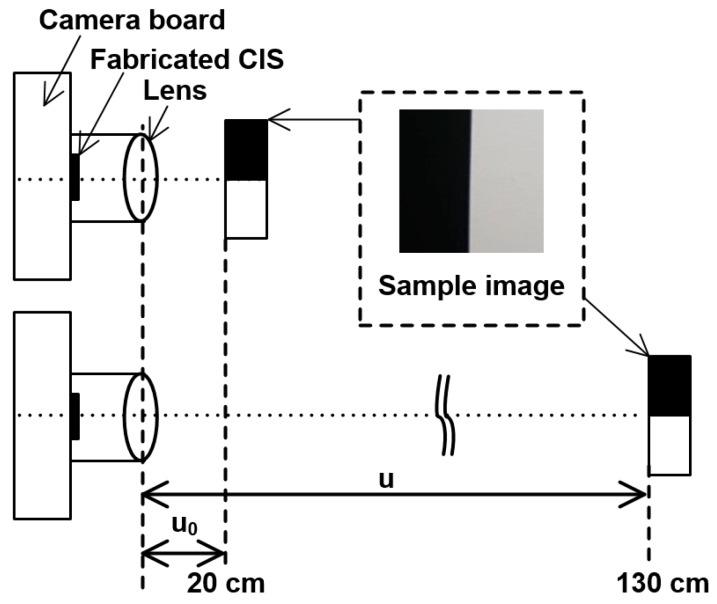
Measurement setup for evaluation of disparity information.

Figure 10 shows the measurement setup for evaluation of the disparity information. The black and white printed image with an edge was used for measurement to calculate disparity. The measurement system comprises a camera board, fabricated CIS, camera lens, and rail to control the distance. The F-number and focal length of the camera were 1.4 and 16 mm, respectively. The camera lens was focused at 20 cm, and the distance between the camera lens and the black-and-white printed image was controlled from 20 cm to 130 cm. The output image of the fabricated CIS, according to the distance, was measured, and the edge of the output images was defined as the point where the light intensity was half the maximum value.

**Figure 11:**
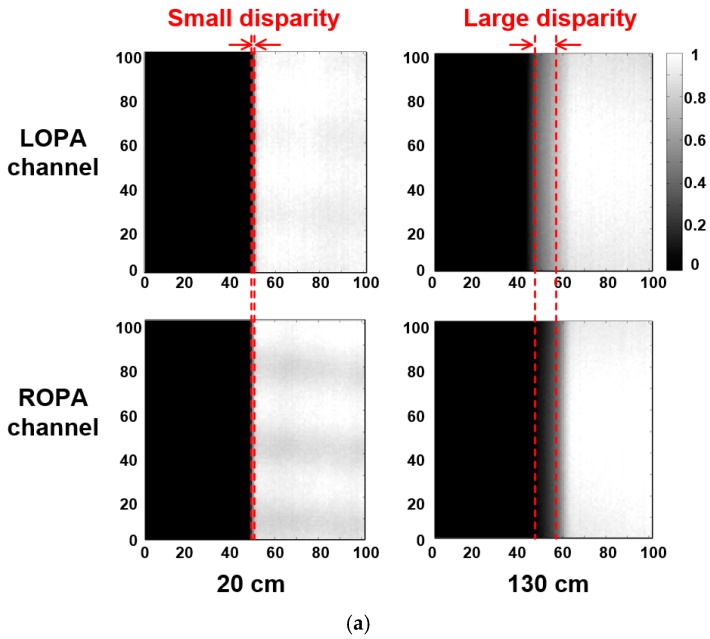

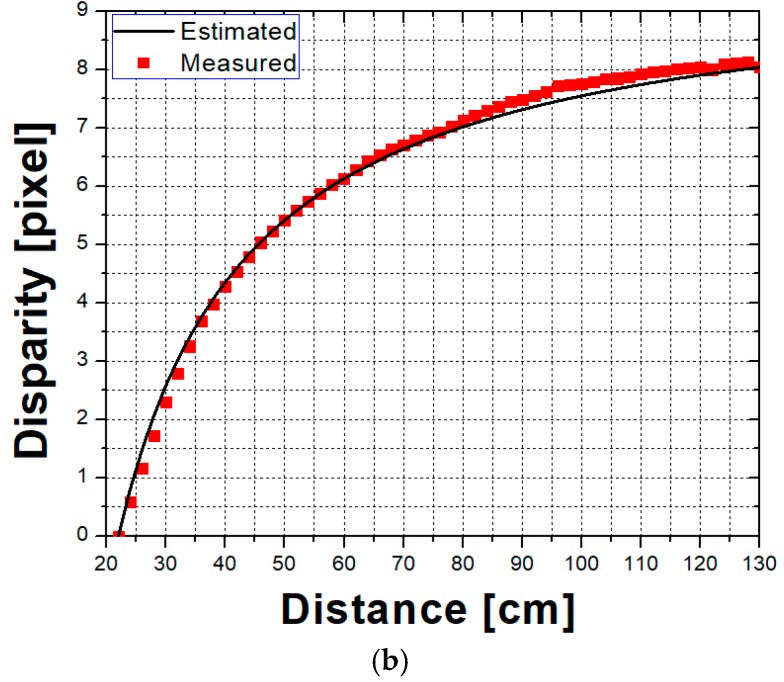
(**a**) Output images from the LOPA and ROPA channels, and (**b**) the measured and estimated results with disparity, according to distance.

The output images from the LOPA and ROPA channels are shown in Figure 11a. The measurement was implemented using the measurement setup in Figure 10. The output images from the LOPA and ROPA channels are separated by the raw image from the LOPA, blue, red, green, and ROPA channels. The output image with 101 × 101 pixels is the central part of the sample image. The output image is also averaged over 100 frames to reduce temporal noise and is normalized. Edge of the image is defined as the point where the intensity drops to half of the maximum [20]. Comparing the disparities, the disparity at the distance of 130 cm is larger than that of 20 cm.

The measured and estimated results with disparity, according to distance, are shown in Figure 11b. The disparity was calculated by subtracting the positions of the edges in the output images from the LOPA and ROPA channels. The disparity increases with distance, and the depth can be calculated from the disparity information. The measurement results with disparity agreed well with the results estimated by Equation (7). The root-mean square difference between the measured and estimated results is only 0.14 pixel. These results confirm that the theory of the OPA technique and measurement are in good agreement.

## 5. Conclusions

A CIS using the OPA technique for depth extraction was fabricated using a 0.11-µm CIS process and developed. The color pattern of the OPA technique comprised the LOPA, blue, red, green, and ROPA pixels. The color information can be obtained by the blue, red, and green pixels, and the disparity information related to the distance between the camera lens and the object was obtained by the LOPA and ROPA pixels. Moreover, the aperture offset in the LOPA and ROPA pixels was optimized by the optical simulation using the finite-difference time-domain method. In the measurement results, the disparity of the LOPA and ROPA pixels, according to incidence angle, was evaluated. A disparity of 35.2° with an aperture offset of 0.65 µm in the LOPA and ROPA pixels was achieved in the simulation result, whereas a disparity of 36.9° was achieved in the measurement result. The measurement results agreed well with the simulation results, except for a small difference caused by the different characteristics between the ideal microlens model for the simulation and the fabricated microlens. Additionally, the disparities according to distance were measured. The measurement results confirmed that disparity increases with the distance and that the root-mean square difference between measured result and estimated result from the OPA model is only 0.14 pixel. The measurement results are in good agreement with the estimated results with the obtained theoretical expressions. This work will be useful for depth extraction using CIS with high resolution and low cost, and the developed CIS using the OPA technique has many applications such as motion detection, gesture recognition, and object reconstruction.

## Figures and Tables

**Table 1:** Summary of the fabricated CIS.

Parameter	Value
Process	0.11-µm 2-poly 4-metal CIS process
Pixel type	Four transistors active pixel sensor with the pinned photodiode
Pixel size	2.8 × 2.8 µm^2^
Pixel array	1632 (H) × 1124 (V)
Color pattern	LOPA, blue, red, green, and ROPA pixels
Chip size	7 mm (H) × 10 mm (V)
Power supply	3.3 V (analog), 1.5 V (analog and digital)
Frame rate	17 fps
